# Chondroitin Sulfate- and Decorin-Based Self-Assembling Scaffolds for Cartilage Tissue Engineering

**DOI:** 10.1371/journal.pone.0157603

**Published:** 2016-06-17

**Authors:** Lourdes Recha-Sancho, Carlos E. Semino

**Affiliations:** Tissue Engineering Laboratory, Department of Bioengineering, IQS School of Engineering, Ramon Llull University, Barcelona, Spain; University of Zaragoza, SPAIN

## Abstract

Cartilage injury and degenerative tissue progression remain poorly understood by the medical community. Therefore, various tissue engineering strategies aim to recover areas of damaged cartilage by using non-traditional approaches. To this end, the use of biomimetic scaffolds for recreating the complex *in vivo* cartilage microenvironment has become of increasing interest in the field. In the present study, we report the development of two novel biomaterials for cartilage tissue engineering (CTE) with bioactive motifs, aiming to emulate the native cartilage extracellular matrix (ECM). We employed a simple mixture of the self-assembling peptide RAD16-I with either Chondroitin Sulfate (CS) or Decorin molecules, taking advantage of the versatility of RAD16-I. After evaluating the structural stability of the bi-component scaffolds at a physiological pH, we characterized these materials using two different *in vitro* assessments: re-differentiation of human articular chondrocytes (AC) and induction of human adipose derived stem cells (ADSC) to a chondrogenic commitment. Interestingly, differences in cellular morphology and viability were observed between cell types and culture conditions (control and chondrogenic). In addition, both cell types underwent a chondrogenic commitment under inductive media conditions, and this did not occur under control conditions. Remarkably, the synthesis of important ECM constituents of mature cartilage, such as type II collagen and proteoglycans, was confirmed by gene and protein expression analyses and toluidine blue staining. Furthermore, the viscoelastic behavior of ADSC constructs after 4 weeks of culture was more similar to that of native articular cartilage than to that of AC constructs. Altogether, this comparative study between two cell types demonstrates the versatility of our novel biomaterials and suggests a potential 3D culture system suitable for promoting chondrogenic differentiation.

## Introduction

Adult articular cartilage lacks an intrinsic capacity to regenerate after trauma or injury due to its avascularity and low biosynthetic activity [1]. Consequently, cartilage defects are degenerative, thus contributing to the development of compromised tissue function and joint disability [2,3]. Current clinical approaches for repairing cartilage defects include a variety of surgical options, such as autologous chondrocyte implantation and microfracture techniques [4–6]. However, these treatments often result in the formation of fibrocartilage tissue with inferior biomechanical properties compared to the original cartilage [7]. Therefore, the development of new strategies to restore and repair damaged areas is of growing interest [8]. In this regard, cartilage tissue engineering (CTE) attempts to create functional substitutes through the appropriate combination of cells, scaffolds and stimulatory factors [9,10].

Candidate cell types for cartilage repair include articular chondrocytes (ACs) and mesenchymal stem cells (MSCs) because chondrocytes already possess the desired phenotype and MSCs present lineage potential to differentiate into mature chondrocytes [11]. In principle, ACs are the only resident cell type in mature articular cartilage and are therefore responsible for the synthesis and remodeling of the extracellular matrix (ECM). Once they are isolated from their natural surrounding matrix and cultured in a monolayer for cell expansion, they undergo dedifferentiation and lose the expression of specific chondrogenic markers, including collagens and glycosaminoglycans [12,13]. Consequently, they acquire a fibroblast-like phenotype, which compromises their use in CTE applications. Nevertheless, promising results have been obtained with different three-dimensional (3D) culture platforms to restore and maintain the chondrogenic phenotype [14–17]. In contrast, MSCs are multipotent progenitor cells that possess the ability to proliferate *in vitro* and differentiate into lineages of mesodermal origin, including bone, cartilage and fat [18,19]. They can be isolated from different sources, such as bone marrow, muscle, adipose tissue and the umbilical cord [20]. In particular, MSCs of adipose origin are easy to acquire and allow an abundant supply of cells with minimally invasive surgery [21]. Along with these reasons, the plasticity of MSCs makes them a promising source of adult stem cells in CTE applications. In this work, expanded dedifferentiated ACs and adipose-derived MSCs, both from human origin, were selected for evaluation in a comparative study of chondrogenic differentiation using specific culture conditions and biomimetic scaffolds [22].

The composition and structure of the ECM govern the physical, biochemical and biomechanical signals that are continuously received by cells [23]. Therefore, biomaterials are designed to mimic the complex cellular microenvironment while providing cells with the appropriate cues [24,25]. Hydrogels are attractive candidates as tissue engineering scaffolds because they are biocompatible and possess a unique hydrated 3D network, thus recreating the nano-architectural pattern of the natural ECM [8]. Importantly, self-assembling peptides provide a network of interweaving nanofibers (50 to 200 nm pore size), which allow cells to experience a truly 3D environment. The self-assembly process is driven by noncovalent interactions (e.g., hydrogen bonds, electrostatic interactions) under physiological conditions, allowing cells to freely extend processes for intercellular interactions, migration and proliferation [26,27]. Moreover, self-assembling peptides are synthetic hydrogels with reproducible, controllable and customizable properties. For these reasons, in the present work 3D cultures were based on the self-assembling RAD16-I peptide (AcN-(RADA)_4_-CNH_2_), which has been widely used to culture various mammalian cell types for their growth and differentiation [28–35]. The mechanical properties of the cultured cells can be controlled by changing the peptide concentration, which enables their use in different tissue engineering applications [36]. The RAD16-I scaffold lacks the intrinsic capacity to instruct cells through receptor/ligand interactions, but it can be modified to incorporate specific signaling motifs or functional molecules [37,38]. In this regard, we have previously shown that noncovalent interactions between the RAD16-I nanofibers and heparin moieties can form a stable bi-component scaffold with growth factor (GF) binding affinity [39]. This finding demonstrates a potential use in vascular and CTE applications because the biomaterial could promote different cellular processes, depending on the conditions provided (cell type, culture media and peptide concentration) [39,40]. To expand on our previous work, the aim of this study was to develop novel biomaterials to support chondrogenesis by taking advantage of the ability of the RAD16-I scaffold to interact with other biomolecules. Our approach was based on mimicking the native articular cartilage ECM while providing bioactive signals to the non-instructive RAD16-I peptide scaffold. Glycosaminoglycans (GAGs) and proteoglycans (PGs) are important structural components of cartilage that influence the regulation of cell proliferation, migration and differentiation [41]. Among them, Chondroitin Sulfate (CS) and Decorin were selected in this work and were separately combined with the self-assembling peptide RAD16-I. CS is a sulfated anionic polysaccharide and GAG constituent of PGs, and Decorin is a small PG that contains a core protein bound to one chain of CS [42,43]. These molecules play several important roles in regulating different cellular responses [44]. For instance, they bind to GFs, such as transforming growth factor-β (TGF-β), which interacts with both the protein core and the side chain of CS [45,46]. We hypothesize that the presence of CS or Decorin in combination with the RAD16-I scaffold could modulate chondrogenesis under different experimental conditions. Two different cell types were cultured with our novel bi-component scaffolds to re-differentiate expanded human chondrocytes and guide MSCs to cartilage commitment.

## Materials and Methods

### Toluidine blue staining

RAD/CS and RAD/Decorin composites were prepared by combining 95 μL of RAD16-I 0.5% (w/v) and 5 μL of chondroitin sodium salt (C3788, Sigma) or Decorin (D8428, Sigma) in a concentration range between 0.01 and 1% (w/v). Control RAD16-I samples were prepared with a final concentration of 0.5% (w/v). First, 100 μL of each sample was loaded into a cell culture insert (PICM-1250, Millipore) in a 6-well culture plate, and 500 μL of PBS was added under the insert to start the self-assembly process. Samples were incubated for 30 minutes at room temperature to allow gelation. Then, 200 μL of PBS was added at the inner wall of the insert, allowing it to slowly slide to the gel, and 2.5 mL of PBS was added outside the insert. Toluidine blue staining was then performed to evaluate the presence of highly negative charges provided by the CS or Decorin molecules. Samples were incubated with toluidine blue 0.05% (w/w) in water for 20 minutes and then washed several times with distilled water. Stained samples were analyzed under a stereoscopic microscope (Nikon SMZ660).

For toluidine blue staining of 3D cell constructs, samples were washed with PBS and then fixed with PFA 2% (w/v) for 1 hour at room temperature. Samples were incubated with toluidine blue 0.05% (w/w) in water for 20 minutes and then washed several times with distilled water. Stained samples were analyzed under a stereoscopic microscope (Leica M165 C).

### Congo red staining

Congo red staining was performed to evaluate the presence of β-sheet structural characteristics of the self-assembling peptide RAD16-I. Samples were incubated with 0.1% (w/v) congo red (75768, Sigma) in water for 5 minutes and washed several times with PBS. The samples were analyzed under a stereoscopic microscope (Nikon SMZ660).

### ELISA quantification of GF release

RAD/CS, RAD/Decorin and RAD/Heparin composite gels were prepared by combining 95 μL of RAD16-I 0.5% (w/v) and 5 μL of 0.2% (w/v) of the corresponding molecule (CS, Decorin or Heparin). Control RAD16-I samples were prepared at a final concentration of 0.5% (w/v). All gels were prepared in triplicate and incubated with a solution of 500 ng/ml TGFβ1 in binding buffer (DMEM, High Glucose, GlutaMAX with 0.1% BSA) for 3 hours at 37°C and 5% CO_2_. Then, the GF solution was removed, and the gels were incubated with binding buffer to allow for the release of TGFβ1. Noncumulative measurements were taken at 12, 24, 36, 60 and 84 hours, which required removing the excess binding buffer containing free GF and adding fresh binding buffer to the gels. Samples were analyzed with ELISA kits for TGFβ1 (ab100647, Abcam) according to the manufacturer’s protocol.

### 2D culture of human ACs and ADSCs

ADSCs (PT-5006, Lonza) (<6th passage) were cultured in 175 cm^2^ flasks in ADSC Basal Medium (ADSC-BM) (PT-3273, Lonza) supplemented with ADSC Growth Medium (ADSC-GM) SingleQuots (PT-4503, Lonza). Cultures were maintained in a humidified incubator at 37°C and 5% CO_2_.

AC cells (CC-2550, Lonza) were cultured at the recommended seeding density (10,000 cells/cm^2^) from passage 2 to passage 6 in 25, 75 and 175 cm^2^ culture flasks. The expansion medium was composed of Chondrocyte Basal Medium (CBM) (CC-3217, Lonza) plus SingleQuots of Growth Supplements (CC-4409, Lonza) containing R3-IGF-1, bFGF, transferrin, insulin, FBS and gentamicin/amphotericin-B. Cultures were maintained in a humidified incubator at 37°C and 5% CO_2_.

### Culture of 3D scaffolds

To obtain 3D cultures, RAD16-I (PuraMatrix™, 354250, Corning) and composite RAD/CS, RAD/Decorin and RAD/Heparin were prepared at a final concentration of 0.3% (w/v) RAD16-I. The composites were prepared by combining 95 μL of RAD16-I 0.5% (w/v) and 5 μL of CS, Decorin or Heparin at a concentration 0.2% (w/v). The mixture was then diluted to a final concentration of 0.3% (w/v) RAD16-I.

The peptide solution was mixed with an equal volume of cell suspension at 4 x 10^6^ cells/mL in 10% sucrose (S0389, Sigma) to obtain a final concentration of 2 x 10^6^ cells/mL in 0.15% (w/v) of RAD16-I and 10% (w/v) sucrose. Then, 80 μL of the cell-peptide mixture (160,000 cells) was loaded into individual wells of a 48-well culture plate previously equilibrated with 150 μL of control or expansion media. Control medium was prepared with DMEM, High Glucose, GlutaMAX (61965, Gibco), ITS+Premix 100x (354352, BD Bioscience), 100 U/mL Penicillin/ 100 μg/mL Streptomycin (P11-010, PAA), 40 μg/mL L-Proline (P5607, Sigma) and 1 mM Sodium Pyruvate (11360, Life Technologies). Upon loading the mixture, the medium induced the self-assembly of RAD16-I, and the cells were homogenously distributed in the scaffold. Then, the plate was incubated for 20 minutes at 37°C and 5% CO_2_, and 650 μL of fresh medium was added to the 3D cell cultures, which were then maintained at 37°C and 5% CO_2_ for 4 weeks. The medium was changed every second day by removing 400 μL from the well and adding 400 μL of fresh medium. Cultures for chondrogenic differentiation were induced at day 2 with chondrogenic medium (control medium supplemented with 10 ng/mL TGFβ1 (GF111, Millipore), 25 μg/mL L-Ascorbic Acid 2-phosphate (A8960; Sigma) and 100 nM Dexamethasone (D8893; Sigma)).

Cultures were maintained for 4 weeks in the described serum-free media under control or chondrogenic conditions (in the presence of stimulating factors to induce chondrogenic differentiation) [47,48]. After 4 weeks of culture, 3D constructs were analyzed for morphology, viability, gene and protein expression, structural characteristics and mechanical properties.

### DAPI and phalloidin staining

3D constructs were washed with PBS, fixed with PFA 2% (w/v) for 1 hour at room temperature and incubated with PBS containing 0.1% Triton X-100 for 30 minutes. Subsequently, samples were stained with 1 μg/mL phalloidin-tetramethylrhodamine B isothiocyanate (phalloidin-TRITC) in PBS for 25 minutes in the dark to visualize the cytoskeleton. Then, they were incubated with 0.1 μg/mL DAPI in PBS for 5 minutes to stain the nuclei and washed with PBS. Samples were analyzed under a Zeiss Axiovert 200M inverted fluorescence microscope with a coupled ApoTome system.

### Cell viability

The live/dead staining procedure was performed by washing the constructs with PBS, incubating with a solution of 2 μM EthD-1 and Calcein AM in PBS (L3224; Invitrogen) for 15 minutes and washing again with PBS. Then, entire constructs were analyzed under a fluorescence microscope to detect live cells (green) and dead cells (red).

### MTT assay for cell viability

Cell viability was assessed using a MTT [3-(4,5-dimethylthiazol-2-yl)-2,5-diphenyltetrazolium bromide] (M5655, Sigma) assay. Briefly, the medium was aspirated and MTT reagent was added to a final concentration of 0.5 mg/mL in culture medium. Samples were incubated for 3 hours at 37°C in the dark. Subsequently, the solution was aspirated, and the constructs were lysed using DMSO (D8418, Sigma). The absorbance was read at 550 nm in a microplate reader (Biotek ELX808).

### Scanning Electron Microscopy (SEM)

After 4 weeks of culture, constructs were fixed in 2% (w/v) PFA and dehydrated in successive ethanol washes. Once dehydrated, samples were dried using a CO_2_ critical point dryer (Emitech K850). Dried samples were subsequently coated with a thin layer of graphite (approximately 40–50 nm) (Emitech K950X). Finally, samples were examined under a JEDL J-7100 field emission scanning electron microscope (Cathodeluminiscence spectrometer GATAN MONO-CL4, EDS detector, retroscattered electron detector) at an accelerating voltage of 15 and 20 kV.

### Real-time reverse transcriptase-polymerase chain reaction (RT-PCR)

RNA was extracted from the samples using a peqGOLD total RNA kit (12-6834-02; PeqLab). After the removal of genomic DNA with a Turbo DNA-free kit (AM1907; Invitrogen), cDNA was synthesized using a High-Capacity cDNA Reverse Transcription Kit (4368814; Applied Biosystems). The cDNA obtained was analyzed by real-time RT-PCR using iQ SYBR Green Supermix (170–8884; Bio-rad) and primers designed for each gene of interest. The primers used were as follows: ribosomal protein L22 (*RPL22*), forward 5’-TGACATCCGAGGTGCCTTTC-3’, reverse 5’-GTTAGCAACTACGCGCAACC-3’; collagen type I (*COL1*), forward 5’-AGACGGGAGTTTCTCCTCGG-3’, reverse 5’-CGGAGGTCCACAAAGCTGAA-3’; collagen type II (*COL2*), forward 5’-ATGACAATCTGGCTCCCAAC-3’, reverse 5’-CTTCAGGGCAGTGTACGTGA-3’; collagen type X (*COL10*), forward 5’-CCAATGCCGAGTCAAATGGC-3’, reverse 5’-GGGGGAAGGTTTGTTGGTCT-3’; aggrecan (*ACAN*), forward 5’-TGGTGATGATCTGGCACGAG-3’, reverse 5’-CGTTTGTAGGTGGTGGCTGT-3’; *SOX9*, forward 5’-CAGACGCACATCTCCCCCAA-3’, reverse 5’- GCTTCAGGTCAGCCTTGCC-3’; human *RUNX2*, forward 5’- GGTTCAACGATCTGAGATTTGTGGG-3’, reverse 5’-CACTGAGGCGGTCAGAGAACAAACTAG-3’. Real-time PCR was carried out under the following conditions: 10 min at 95°C followed by 40 cycles of 15 s at 94°C, 30 s at 55°C (for *COL2* and *RUNX2*) or 60°C (for *RPL22*, *COL1* and *COL10*) or 62°C (for *SOX9*) or 64°C (for *ACAN*), and 30 s at 72°C. Finally, a melting step was performed from 58°C to 95°C to obtain the melting curve. Relative gene-fold variations were determined according to the 2^-ΔΔCt^ method using the ribosomal protein L22 as a housekeeping gene.

### Western blot

Samples were lysed in RIPA buffer (R0278; Sigma), with a protease inhibitor cocktail (Complete Mini, 11836153001; Roche). Acrylamide gels were prepared according to the size of the proteins, generally at concentrations of 7.5% or 10% (w/v). Cell lysates (5 mg of each sample) were run by applying 150 V for 90 min. After migration through the gel, proteins were transferred to a polyvinylidene difluoride (PVDF) membrane (LC 2005; Invitrogen) by applying 40 V for 2 hours at room temperature. The membrane was incubated at room temperature for 2 hours in blocking buffer (BB) consisting of 4% (w/v) nonfat milk powder in PBST. Membranes were incubated for 1 hour at room temperature with primary antibodies at a final concentration of 1 mg/mL in PBST. Then, a species-specific immunoglobulin G-horseradish peroxidase (IgG-HRP) secondary antibody was added, at a final concentration of 1 mg/mL, and incubated at room temperature for 1 h. Finally, the membrane was evaluated for HRP detection with SuperSignal West Pico Chemiluminescent Substrate (34080; Thermo Scientific). Chemiluminescent images were taken in the ImageQuantTM LAS 4000 mini (GE HealthCare). Anti-Actin (sc-1615; SCBT), anti-Collagen I (ab138492; Abcam), anti-Collagen II (ab3092; Abcam) and anti-Collagen X (ab182563; Abcam) were used as primary antibodies. Anti-goat IgG-HRP (ab97100; Abcam), anti-mouse IgG-HRP (ab97023; Abcam) and anti-rabbit IgG-HRP (ab97051; Abcam) were used as secondary antibodies.

### Von Kossa staining

Von Kossa staining was performed to detect matrix mineralization. 3D constructs were washed with PBS and fixed with PFA 2% (w/v) in PBS for 1 hour at room temperature. Then, cultures were washed several times with distilled water to completely remove the PBS to prevent precipitation with the silver nitrate solution. Then, cultures were incubated for 1 hour with a solution of 5% (w/v) silver nitrate (209139, Sigma) in a dark chamber. The culture was then washed several times with distilled water and placed under a bright light source for 10 minutes. Finally, samples were analyzed under a stereoscopic microscope (Leica M165 C).

### Dynamic mechanical analysis

The mechanical properties of the 3D constructs cultured with chondrogenic medium were analyzed by Dynamic Mechanical Analysis (DMA) after 4 weeks of culture. A compression assay that used both *DMA Multi-Frequency-Strain* mode and a *frequency sweep* test was applied to each 3D construct with a DMA Q800 (TA Instruments). The conditions of the assay were as follows: Amplitude = 1 μm, Preload force = 0.01 N and Frequency = 1 Hz. The frequency was selected based on the standard working frequency historically used in this type of experiment, and the amplitude was selected based on a range of amplitude values wherein the sample remained constant. Under the same conditions, calf and chicken native cartilage could also be measured. However, the soft nature of the 3D constructs cultured in control medium, constructs cultured for only a few days or the scaffold alone did not allow mechanical measurements under the same conditions. Therefore, only chondrogenic 3D constructs could be compared to native cartilage under the experimental conditions described.

The results were obtained with *TA Instrument Explorer* software and analyzed with *TA Universal analysis* software. The storage modulus (G’), loss modulus (G”), complex modulus (G*) and tan(delta) values were obtained and presented in separate graphics. G’ is the measure of the sample’s elastic behavior, G” measures the viscous response of the material, G* is the sum of both components and tan(delta) is the ratio of the loss to the storage, representing a measure of the energy dissipation of the material.

### Statistics

Samples were prepared in triplicate for the conditions analyzed. All values are expressed as the mean ± SD. Significant differences were analyzed using GraphPad Prism 6. Statistical analysis was carried out by one‐way or two‐way ANOVA, as appropriate, followed by Tukey post-hoc analysis.

## Results

### Chemical and structural characterization of the bi-component scaffolds

**Fig 1:**
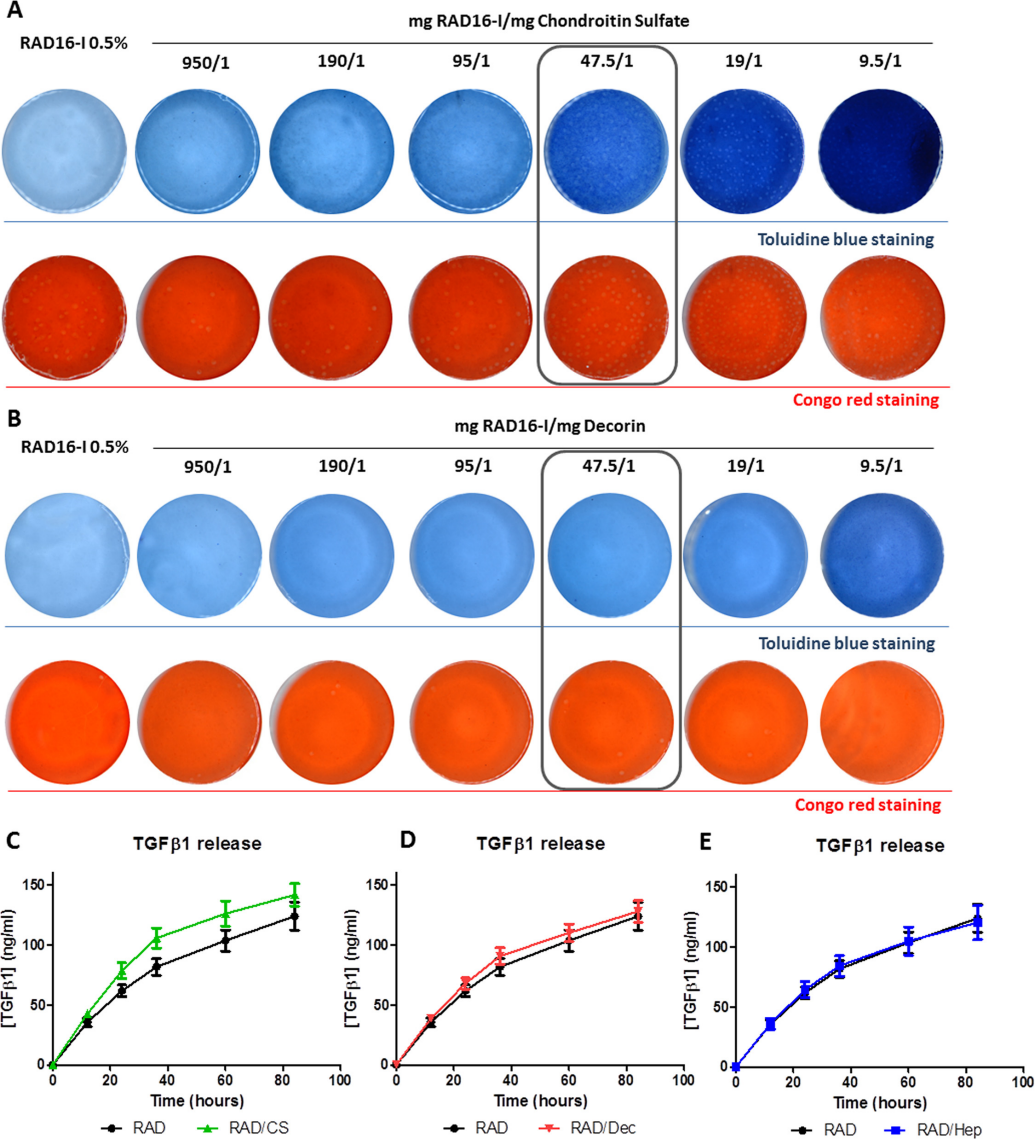
Characterization of the bi-component scaffolds. (A) Toluidine blue and congo red staining of RAD16-I and composites with increasing quantities of CS. Ratios of mg RAD16-I/mg Chondroitin Sulfate ranging from 950/1 to 9.5/1. (B) Toluidine blue and congo red staining of RAD16-I and composites with increasing quantities of Decorin. Ratios of mg RAD16-I/mg Decorin ranging from 950/1 to 9.5/1. (C) Quantification of TGFβ1 released by RAD16-I and the composite RAD/CS (ratio 47.5/1) after 12, 24, 36, 60 and 84 hours of delivery (mean ± SD, n = 3). (D) Quantification of TGFβ1 released by RAD16-I and the composite RAD/Decorin (ratio 47.5/1) after 12, 24, 36, 60 and 84 hours of delivery (mean ± SD, n = 3). (E) Quantification of TGFβ1 released by RAD16-I and composite RAD/Heparin (ratio 47.5/1) after 12, 24, 36, 60 and 84 hours of delivery (mean ± SD, n = 3).

In the present work, CS and Decorin were combined separately with the self-assembling peptide RAD16-I to develop novel scaffolds for CTE applications. Building from previous work with a RAD16-I/Heparin bi-component scaffold [39,40], the chemical and structural stability of the new composites were evaluated by combining different ratios of RAD16-I:CS and RAD16-I:Decorin. As expected, mixtures ranging from 950/1 to 9.5/1 for each composite type were structurally stable at a physiological pH and formed nanofiber composite self-assembling scaffolds (Fig 1A and 1B). Toluidine blue staining was performed to detect highly anionic charged molecules in CS and Decorin. The homogeneous blue color observed in the composite gels after staining confirmed that both CS and Decorin were stably associated to the self-assembling nanofiber network in a dose-dependent manner. As expected, the RAD16-I/Decorin composite showed a less intense blue staining because Decorin is composed of only one single chain of CS covalently bound to a small protein. Moreover, congo red staining showed the formation of β-sheet secondary structures for all scaffolds, thus indicating the proper formation of nanofibers. Therefore, CS and Decorin did not interfere in the self-assembling process, regardless of the concentration. In view of these results, we selected the intermediate ratio of 47.5/1 for both scaffolds (RAD16-I/CS and RAD16-I/Decorin) for further characterization and *in vitro* analysis.

Our next step was focused on studying the GF binding affinity of our biomaterial composites. We selected TGFβ1 as a model GF to evaluate its release profile by the different scaffolds because this GF has an important role in chondrogenic differentiation [47]. To this end, we incubated the new composites (RAD/CS, RAD/Decorin) and the previously described RAD/Heparin composite [39,40] in the presence of TGFβ1 to study its binding and release over time (Fig 1C–1E). In general, the release pattern of TGFβ1 was similar for all tested composites, and these composite scaffolds showed a release pattern similar to that of the control RAD16-I scaffold. However, we observed some differences between the composites. Interestingly, in the case of RAD/CS, more TGFβ1 was released at 24 and 36 hours compared to the control scaffold (Fig 1C), but no differences in GF release were detected between the RAD/Decorin and control RAD16-I scaffold over time (Fig 1D). Finally, as previously reported, a similar TGFβ1 release profile was observed between RAD16-I and the composite RAD/Heparin scaffold, as indicated by the overlapping curves (Fig 1E).

### Induction of chondrogenic differentiation by the bi-component scaffolds

The capacity for inducing chondrogenic differentiation was assessed for RAD16-I/CS, RAD16-I/Decorin and the RAD16-I scaffold alone using two different human cell types: expanded de-differentiated ACs and ADSCs. The aim of the work was to corroborate the versatility of the scaffolds in two different tissue engineering scenarios: differentiation of expanded ACs to their original phenotype and induction of MSCs to a chondrogenic lineage commitment. Cells were seeded in the two different composite scaffolds and maintained for 4 weeks in control or chondrogenic medium (see *Materials and Methods*). Moreover, ACs were cultured in a third medium containing GFs (expansion medium used in monolayer cultures, see Materials and Methods) because this culture condition could affect the fate of the 3D culture.

**Fig 2:**
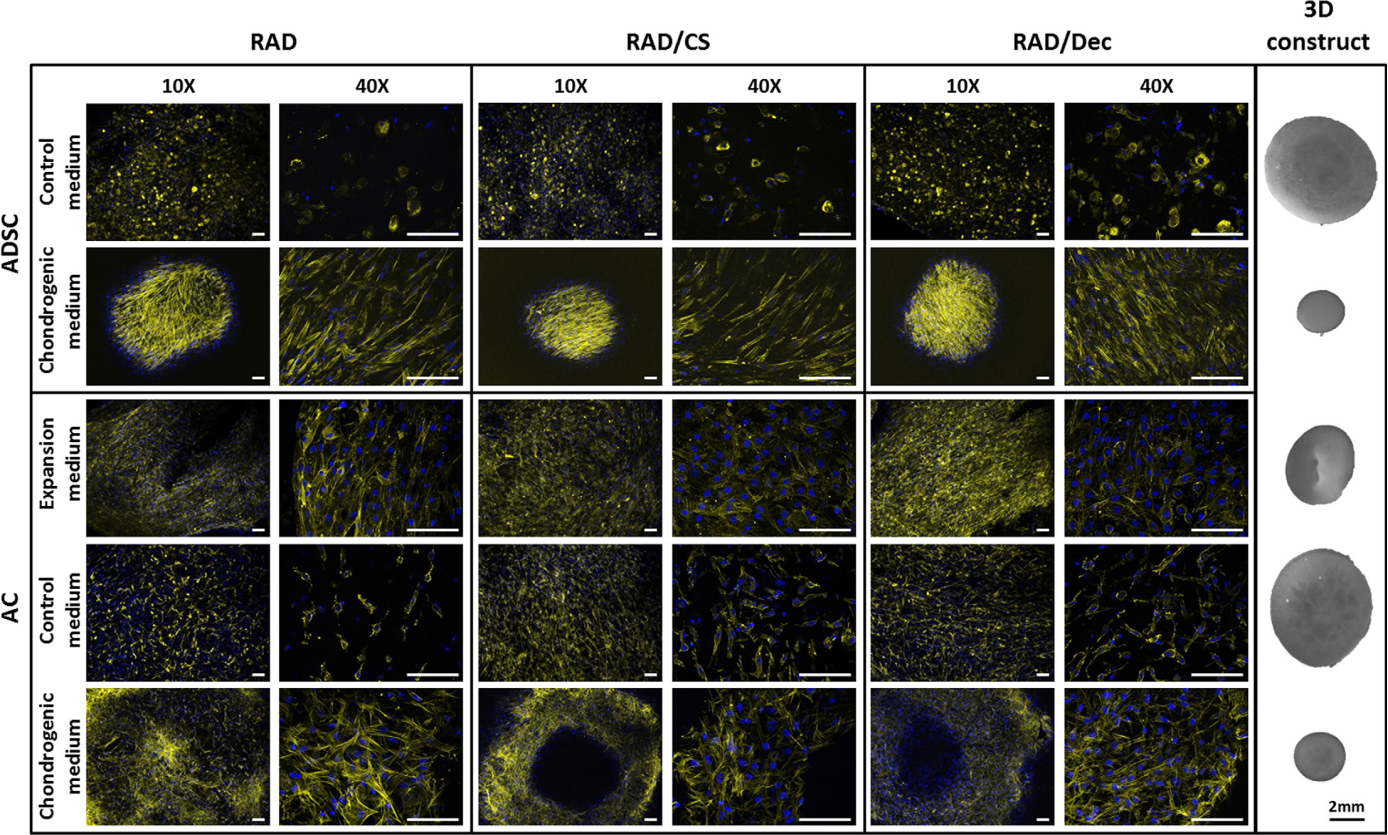
Human ADSCs and ACs cultured under different media conditions with the self-assembling RAD16-I peptide scaffold and bi-component composites. ADSCs and ACs were encapsulated in the control scaffold (RAD16-I) and in the composites (RAD/CS and RAD/Decorin), maintained for 4 weeks in the different media compositions and evaluated throughout the culture period for cell and construct morphology by phase contrast images. Images of 3D constructs show a contracted structure under chondrogenic culture conditions. Fluorescent images of DAPI and phalloidin staining of the three scaffolds after 4 weeks of culture in different culture media (Scale bars = 100 μm).

First, cell morphology was evaluated by DAPI-Phalloidin staining of the cells cultured under the different experimental conditions (Fig 2). In general, good performance was observed in the two cell types for all conditions (with the exception of control), as evidenced by the formation of cellular networks. ADSCs possessed a round morphology under control conditions and were elongated and aligned under chondrogenic conditions. In contrast, ACs were elongated and interconnected in all cases, but lower density cells were observed in control conditions. In addition, construct morphologies were similar between the scaffold types cultured in the same medium; representative images for each condition are shown in Fig 2. Chondrogenic medium causes the most relevant morphological change in both cell types; a reduction in diameter of approximately 70% (compared to diameter at day 0) was observed after 4 weeks of culture. This event correlated with a dense and compacted cellular network observed by DAPI-Phalloidin staining. In contrast, when cultured in control medium, the diameter of constructs was reduced by only a marginal amount from the initial state. A reduction of approximately 50% was observed when ACs were cultured under expansion medium. Therefore, depending on the culture medium, cells developed different construct morphologies.

**Fig 3:**
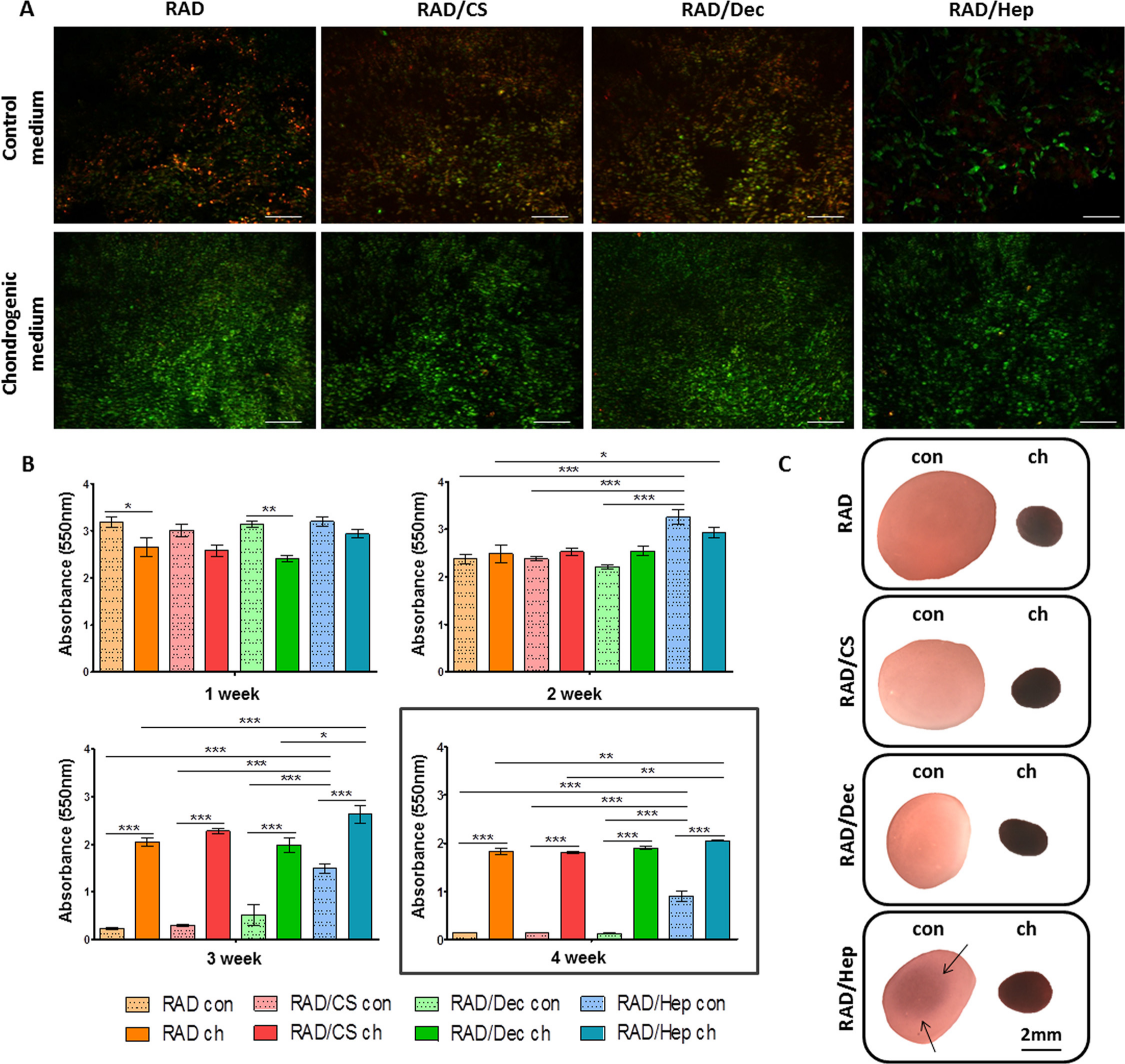
Viability of human ADSCs cultured with control and chondrogenic media in the self-assembling RAD16-I peptide scaffold and in RAD/CS, RAD/Decorin and RAD/Heparin composites. (A) Fluorescent images of live/dead staining at week 4 of culture. Live cells are stained in green and dead cells in red (Scale bars = 200 μm). (B) MTT absorbance values of 3D constructs in both control and chondrogenic culture media in the four scaffold types at different weeks of culture (Significant differences are indicated as * for p<0.05, ** for p<0.01, and *** for p<0.001, One-way ANOVA, N = 2 n = 3). (C) Construct appearance after MTT incubation at week 4 of culture with the different culture media (Con, control medium; ch, chondrogenic medium). Constructs under chondrogenic medium were completely purple after MTT incubation, and constructs under control medium were faintly stained. In the case of RAD/Heparin constructs, live cells were detected in the inner part of the construct (fine arrows).

**Fig 4:**
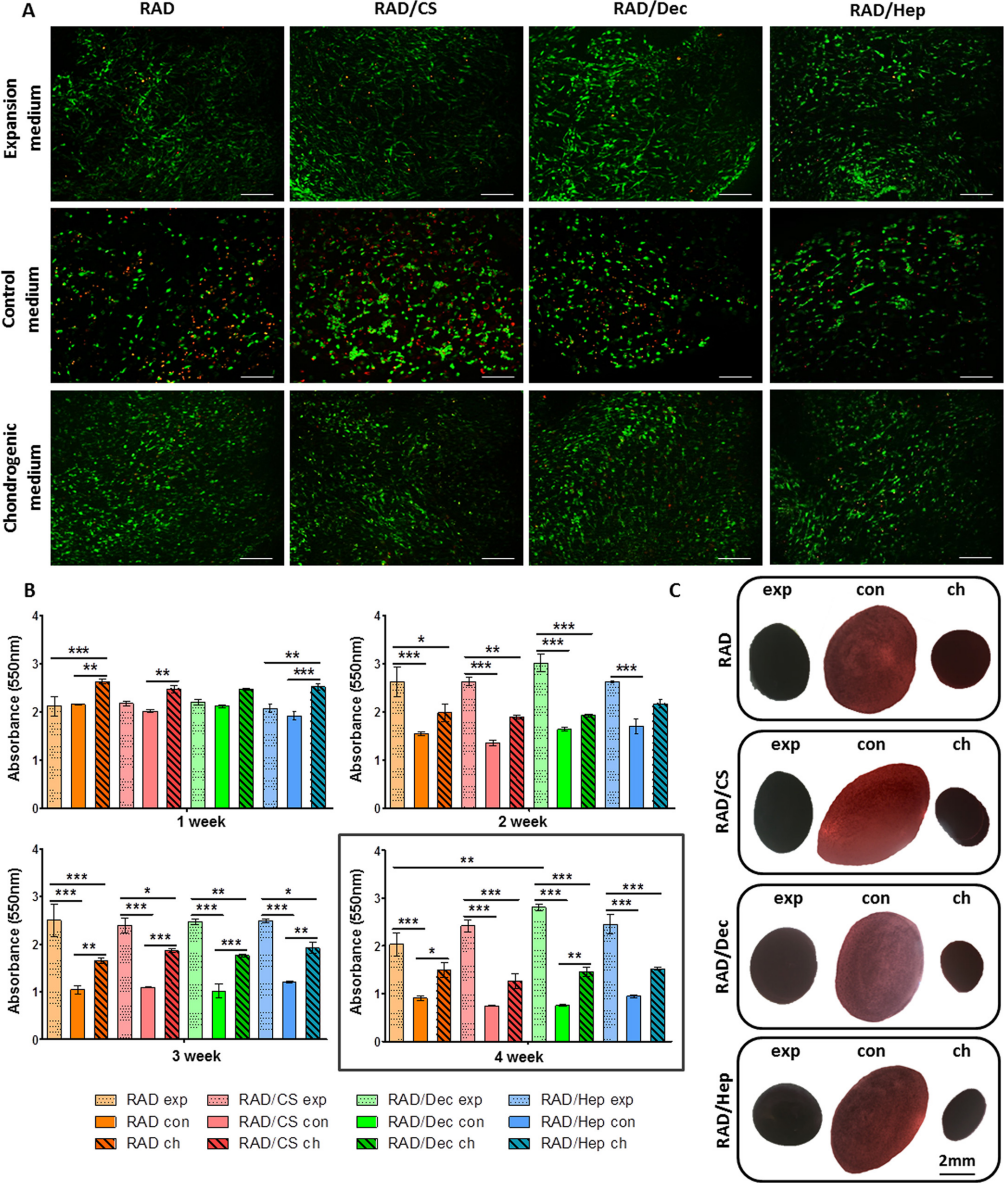
Viability of human ACs cultured with expansion, control and chondrogenic media in the self-assembling RAD16-I peptide scaffold and in RAD/CS, RAD/Decorin and RAD/Heparin composites. (A) Fluorescent images of live/dead staining at week 4 of culture. Live cells are stained in green and dead cells in red (Scale bars = 200 μm). (B) MTT absorbance values of 3D constructs in the three culture media in the four scaffold types at different weeks of culture (Significant differences are indicated as * for p<0.05, ** for p<0.01, and *** for p<0.001, One-way ANOVA, N = 2 n = 3). **(C)** Construct appearance after MTT incubation at week 4 of culture with the different culture media: expansion (exp), control (con) and chondrogenic (ch) media.

Cellular viability in the 3D cultures was assessed by a quantitative MTT assay at different time points throughout the culture period and by qualitative live/dead staining at the end of the culture (Figs 3 and 4). Interestingly, ADSCs that were cultured in chondrogenic medium remained alive until the end of the culture period, whereas the majority of ADSCs cultured in control medium died by the end of the culture period, with the exception of those cultured with RAD/Heparin composites (Fig 3A). This finding is consistent with a previous study [39] in which the presence of heparin in the scaffold promoted ADSC viability, but this phenomenon was not observed in the case of the CS or Decorin scaffolds. Furthermore, the viability of cells cultured with different constructs was similar during the first 2 weeks of culture, but drastic cell death occurred during the third week for samples incubated in control medium (Fig 3B and 3C). Remarkably, constructs cultured with RAD/Heparin composites showed significantly higher viability over the experimental timeframe. Viable cells cultured in control medium with RAD/Heparin composites were detected mainly in the inner area of the constructs (Fig 3C).

A different behavior was observed for AC cells in the 3D constructs. Cells remained predominantly alive in all experimental conditions by week 4 of culture, regardless of the culture medium or scaffold type (Fig 4A). Although some dead cells could be detected in constructs cultured in control medium, the majority of cells were alive. In the two other culture media (expansion and chondrogenic), cells appeared more compact compared to control medium. Moreover, viability profiles along the culture showed increasing differences between culture media over time (Fig 4B). At week 1 of culture, viability was maintained at similar levels between construct types, and some differences could be detected between chondrogenic constructs compared to the other culture media conditions. Through 2 weeks of culture, constructs under expansion medium presented significantly higher viability than did those under control and chondrogenic media. A similar tendency was observed at 3 weeks of culture; however, in addition, significant differences were detected between control and chondrogenic constructs. Therefore, at the end of the culture period, the constructs in expansion medium showed the highest absorbance values, those cultured in control medium showed the lowest values, and those under chondrogenic medium showed intermediate values between those of control and expansion media. These differences are also indicated by the construct’s appearance after MTT incubation at week 4 (Fig 4C). In this case, the presence of heparin in the scaffold did not lead to a significant enhancement in viability of ACs [40].

**Fig 5:**
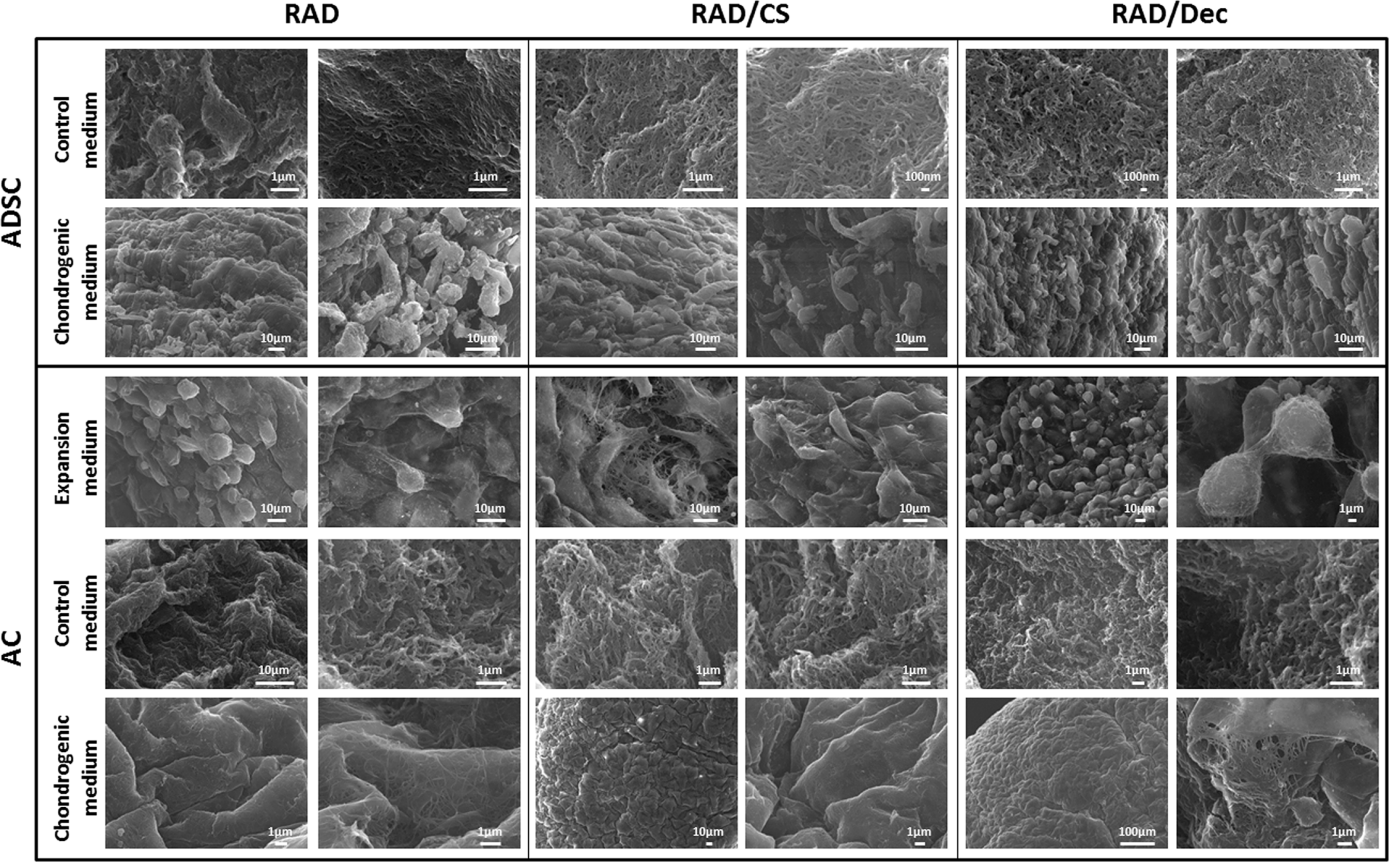
SEM images of ADSCs and ACs cultured in 3D scaffolds after 4 weeks. Cells were seeded into RAD16-I, RAD/CS or RAD/Decorin scaffolds. ADSCs were cultured with control or chondrogenic media; ACs were cultured with expansion, control or chondrogenic media. Two images per condition are shown.

SEM images were collected to more precisely assess cell morphology and the appearance of the surface constructs at week 4 of culture (Fig 5). ADSCs cultured in chondrogenic medium appeared elongated and well-anchored to the scaffold surface. However, SEM images of ADSC constructs in control medium showed nanofibers and other possible ECM components synthesized by the cells during the culture period. In contrast, ACs cultured in expansion medium possessed a spherical shape with possible cell-matrix interactions and thorough ECM components. Similar to ADSCs, nanofibers and putative matrix components could be observed on the surface of constructs cultured in control medium. Additionally, grooves with visible fibers were detected on the entire surface of constructs cultured in chondrogenic medium, suggesting the presence of secreted matrix components. Although cells were not visualized on the surface of the scaffold in all experimental conditions, we hypothesize that they were present in the inner area of the scaffold, as observed by DAPI-Phalloidin staining (Fig 2).

### Expression of chondrogenic markers

**Fig 6:**
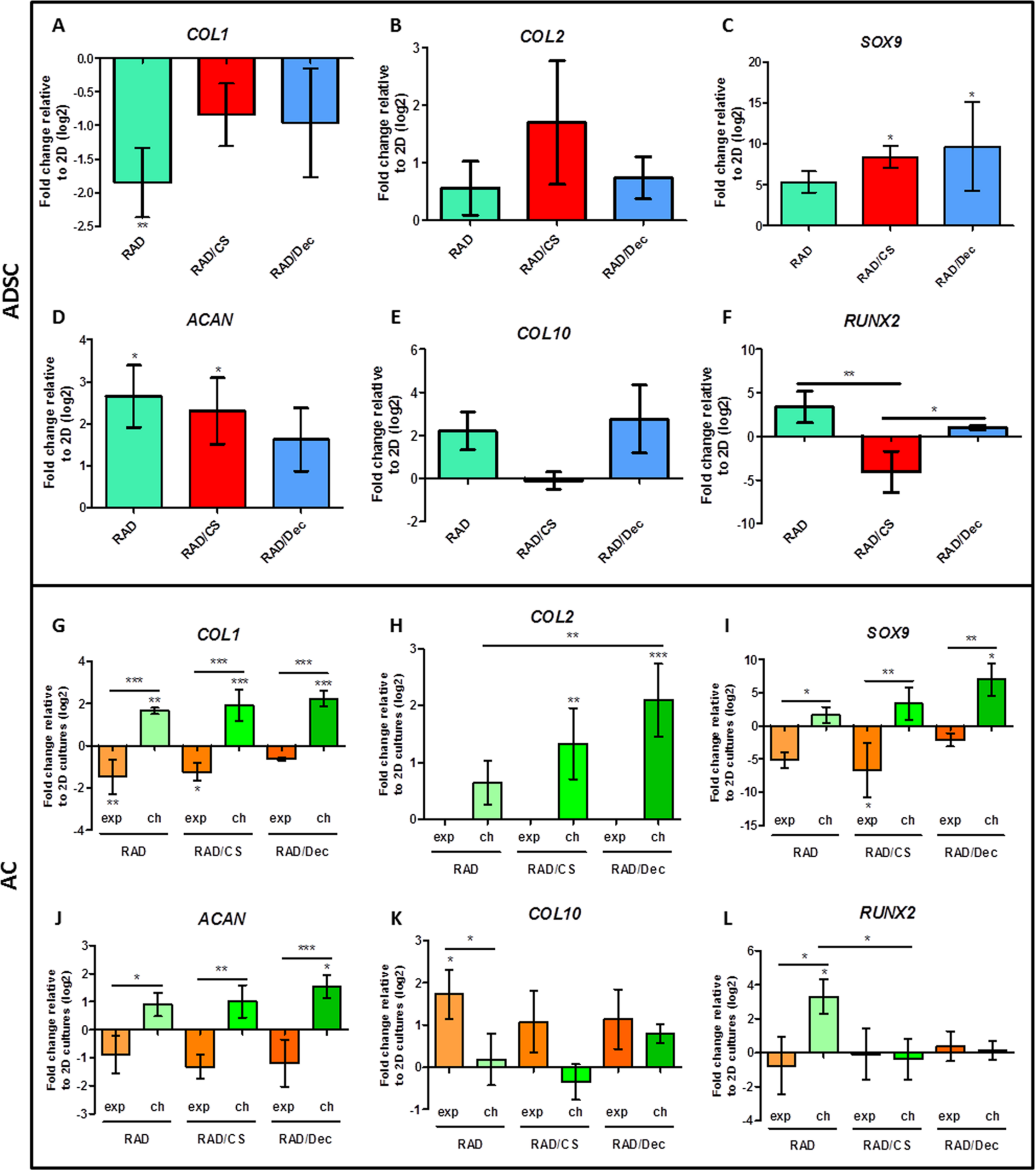
Gene expression levels of chondrogenic and hypertrophic markers of ADSCs and ACs cultured in 3D scaffolds for 4 weeks. ADSCs cultured with RAD16-I, RAD/CS and RAD/Decorin scaffolds in chondrogenic medium were analyzed by qRT-PCR for collagen type I (*COL1*, A), collagen type II (*COL2*, B), *SOX9* (C), aggrecan (*ACAN*, D), collagen type X (*COL10*, E) and *RUNX2* (F). ACs cultured with RAD16-I, RAD/CS and RAD/Decorin scaffolds in expansion (exp) and chondrogenic (ch) medium were analyzed by qRT-PCR for *COL1* (G), *COL2* (H), *SOX9* (I), *ACAN* (J), *COL10* (K) and *RUNX2* (L). Ct values relative to ribosomal protein L22 (RPL22) were obtained and reported as the fold increase (ΔΔCt) relative to 2D cultures (Significant differences are indicated as * for p<0.05, ** for p<0.01, and *** for p<0.001, One-way ANOVA, N = 2 n = 3).

Because no significant differences in cell morphology and viability were detected between CS or Decorin scaffold types, further assessments of gene and protein expression were performed for both cell types. Chondrogenic markers were studied in ADSC constructs cultured in chondrogenic medium and in AC constructs cultured in chondrogenic and expansion media (cell viability was compromised under control medium). Gene expression analyses of different ECM components and transcription factors were analyzed quantitatively, and 3D cultures were compared with their 2D counterparts (Fig 6). In the case of ADSC constructs cultured under chondrogenic medium, the expression of collagen type I (*COL1*) was significantly downregulated in RAD16-I scaffolds and maintained at 2D culture levels in RAD/CS and RAD/Decorin composites (Fig 6A). Collagen type II (*COL 2*) appeared to be upregulated in 3D cultures, but no significant differences were detected (Fig 6B). In contrast, the transcription factor *SOX9*, a regulator of *COL2* expression, was clearly upregulated for all composites (Fig 6C). The characteristic PG of articular cartilage, aggrecan, was significantly upregulated in the RAD16-I scaffold and the RAD/CS composite (Fig 6D). In our analysis of hypertrophic markers, we found that the expression levels of collagen type X (*COL10*) and the transcription factor *RUNX2* in 3D cultures were maintained at levels comparable to 2D culture conditions (Fig 6E and 6F). On the other hand, AC constructs were analyzed in expansion and chondrogenic media, and significant differences could be observed between them. *COL1* was upregulated in 3D constructs under chondrogenic medium and downregulated under expansion medium (Fig 6G). Remarkably, the expression of *COL2* was only upregulated in RAD/CS and RAD/Decorin scaffolds under chondrogenic medium (Fig 6H). As expected, this finding correlates with the expression of *SOX9*, which was significantly upregulated in chondrogenic constructs when compared to 3D constructs cultured in expansion medium (Fig 6I). The expression of *ACAN* was higher in constructs cultured in chondrogenic medium than in constructs cultured in expansion medium (Fig 6J). No significant differences were detected in the expression of hypertrophic markers between 3D constructs compared to monolayer growth conditions, except in the case of the RAD16-I scaffold, in which *COL10* and *RUNX2* expression was upregulated in expansion and chondrogenic media, respectively (Fig 6K and 6L). Therefore, as expected, chondrogenic medium is more effective than expansion medium at promoting chondrogenesis in AC constructs.

**Fig 7:**
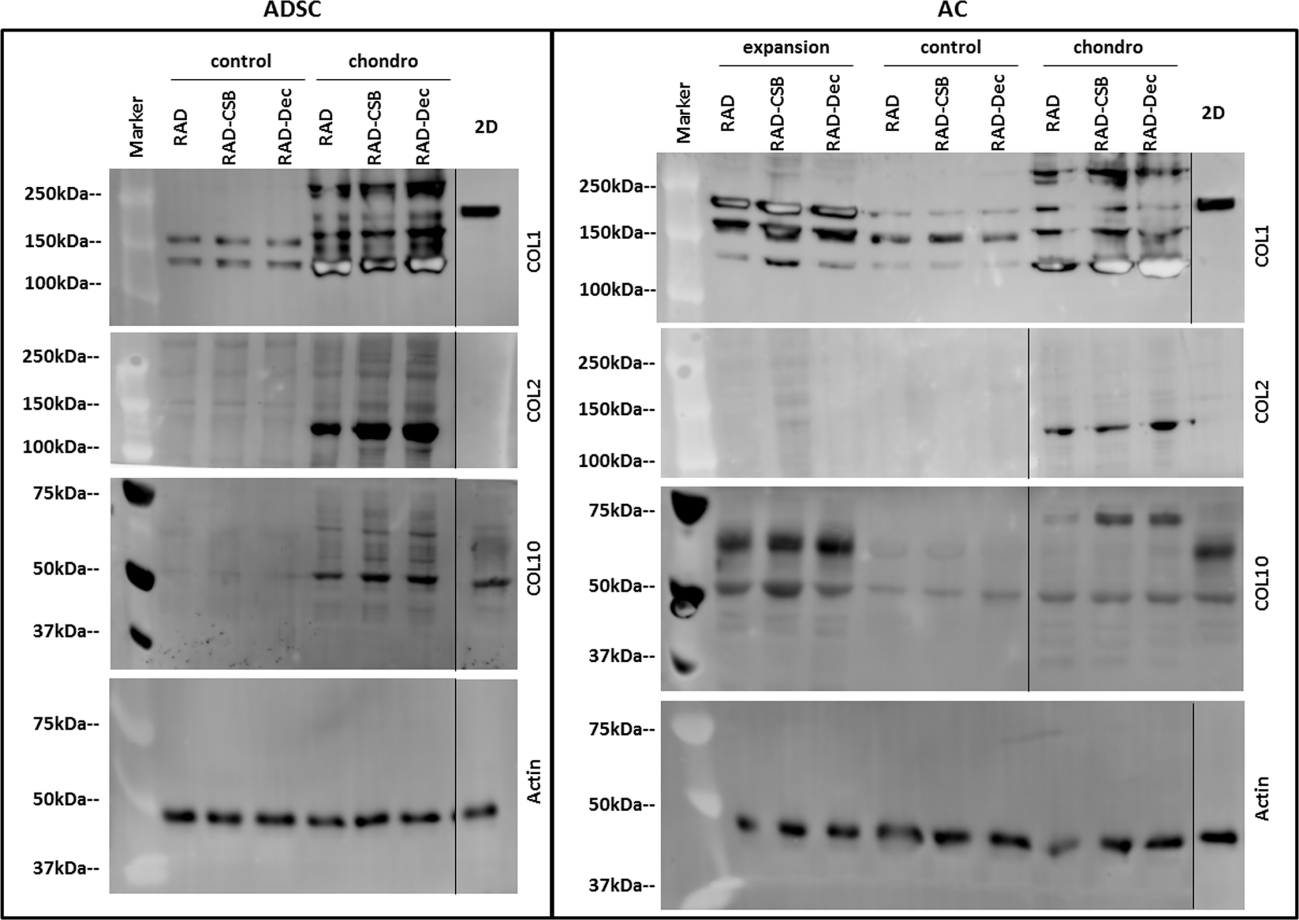
Characterization of protein expression in ADSCs and ACs cultured as monolayers and in 3D cultures after 4 weeks of culture. Western blot results of collagen type I (COL1), collagen type II (COL2) and collagen type X (COL10) from ADSCs (A) and ACs (B) cultured in RAD16-I alone, RAD/CS or RAD/Decorin. Actin was used as an internal control. Samples were prepared in triplicate. Exp, expansion medium; con, control medium; ch, chondrogenic medium.

The protein expression profiles of different collagen constituents of the ECM (collagen type I, II and X) were analyzed by western blot in 2D and 3D cultures of ADSCs and ACs at week 4 of culture (Fig 7). In the case of ADSCs, only the constructs cultured in chondrogenic medium were analyzed because ADSCs cultured in control medium were dead by the end of the culture period (Fig 3). COL1 was detected in both cell types when grown as monolayers or in 3D constructs, but interestingly, different band patterns were observed. In 2D cultures, only a single band of high molecular weight was detected (~220 kDa), which was likely generated by a pro-collagen intermediate. In addition, more bands of lower molecular weight (ranging from 180 to 130 kDa) were observed in 3D cultures. Nevertheless, the intensities of the bands were different between culture medium; for instance, the ~130 kDa band was predominant in 3D constructs cultured in chondrogenic medium. In the case of AC constructs cultured in expansion medium, higher molecular weight bands (~220 kDa and ~180 kDa) presented as more intense than the ~130 kDa band. Importantly, COL2 was detected only in 3D constructs cultured with chondrogenic medium for both ADSCs and ACs, which is consistent with the gene expression results (Fig 6B and 6H). COL10 protein expression was observed in all of the analyzed samples, including the 2D and 3D cultures of both cell types; however, only faint bands were detected in constructs cultured in control medium.

**Fig 8:**
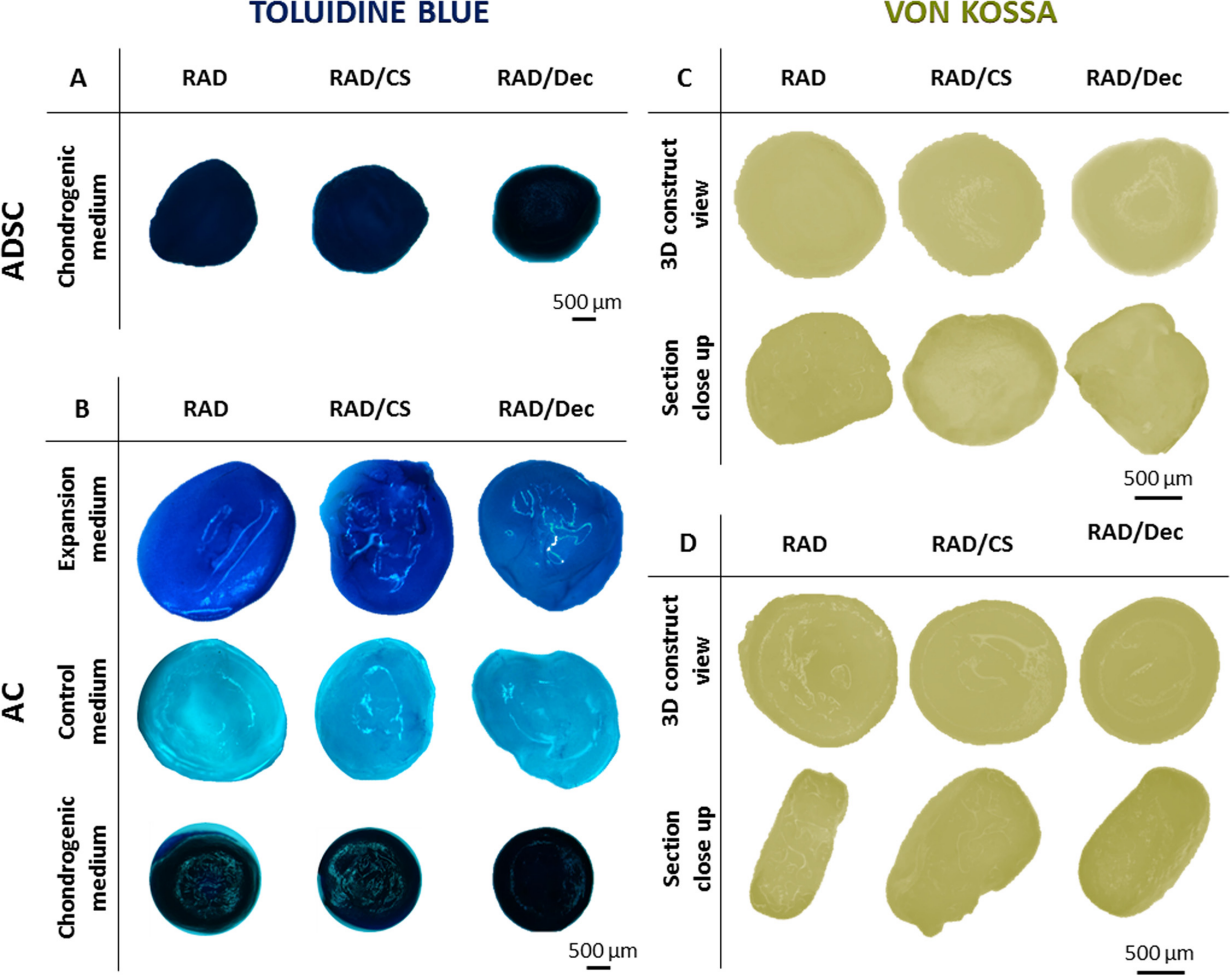
Characterization of chondrogenic phenotypes of ADSCs and ACs cultured with RAD16-I, RAD/CS, or RAD/Decorin composite scaffolds for 4 weeks. (A) Toluidine blue staining (sulfated GAGs) of 3D ADSC constructs cultured in chondrogenic medium. (B) Toluidine blue staining of 3D AC constructs cultured in expansion, control and chondrogenic media. (C) Von Kossa staining (indicating calcium mineralization) of 3D ADSC constructs cultured in chondrogenic medium. (D) Von Kossa staining of 3D AC constructs cultured in chondrogenic medium.

Furthermore, entire 3D constructs were stained with toluidine blue to qualitatively assess the production of GAGs by the cells (Fig 8A and 8B). In both cell types, constructs cultured in chondrogenic medium showed intense blue staining, indicating a significant production and accumulation of GAGs. In the case of ACs, constructs cultured in expansion media showed less GAG staining than did those cultured in chondrogenic medium, whereas constructs cultured with control medium showed only relatively weak staining. Moreover, Von Kossa was performed to assess the production of calcium mineral deposits characteristic of osteogenic differentiation in 3D ADSC and AC constructs cultured in chondrogenic medium (Fig 8C and 8D). As expected, constructs showed no calcification, as indicated by a lack of dark spots detected in external and internal sections.

### Mechanical characterization of tissue constructs

**Fig 9:**
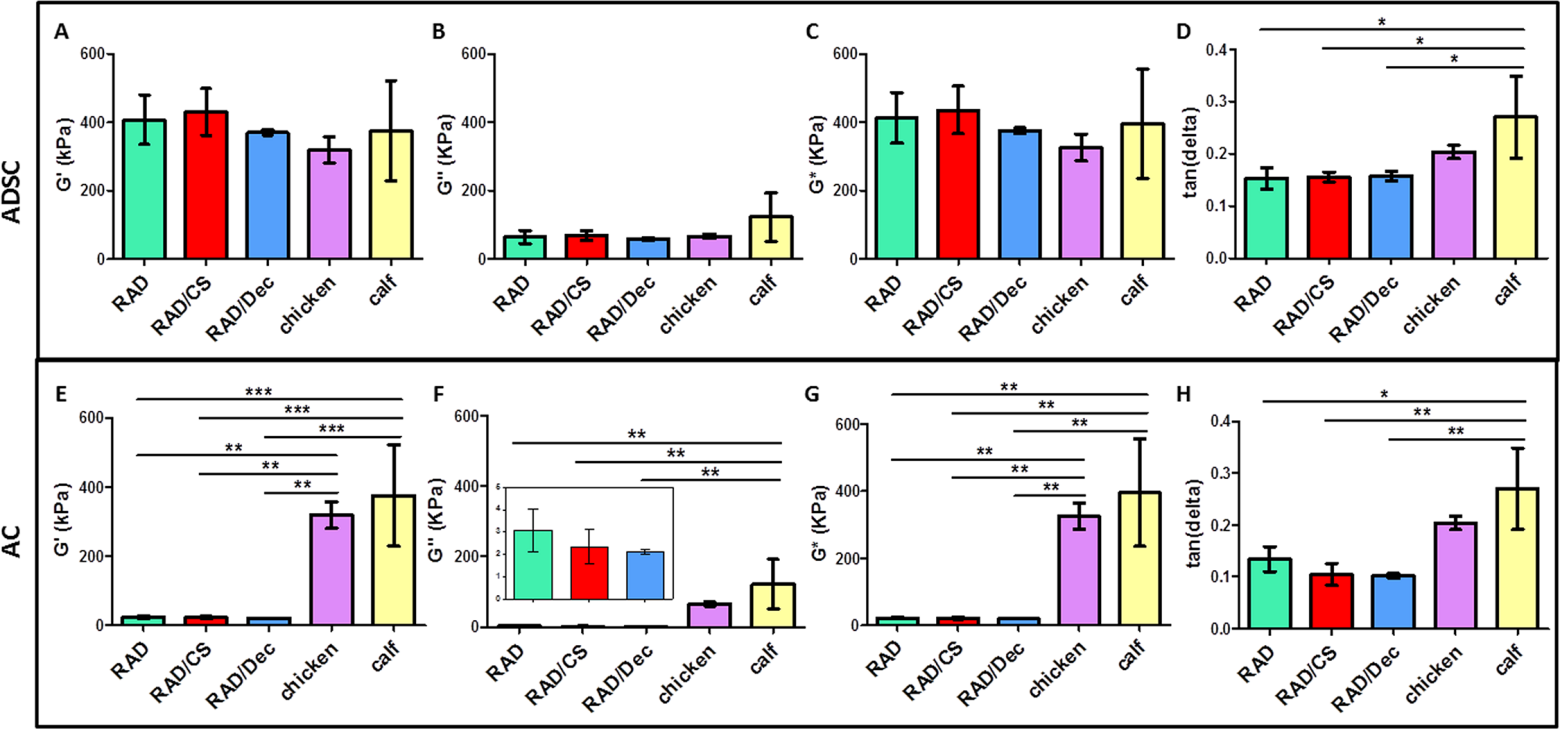
Mechanical characterization of 3D constructs cultured for 4 weeks in chondrogenic medium compared to chicken and calf articular cartilage. ADSCs cultured with RAD16-I, RAD/CS and RAD/Decorin scaffolds were analyzed for storage modulus (G’, A), loss modulus (G”, B), complex modulus (G*, C) and tan(delta) (D). ACs cultured with RAD16-I, RAD/CS and RAD/Decorin scaffolds were analyzed for storage modulus (G’, E), loss modulus (G”, F), complex modulus (G*, G) and tan(delta) (H). (Significant differences are indicated as * for p<0.05, ** for p<0.01, and *** for p<0.001, One-way ANOVA, N = 2 n = 3).

The mechanical properties of both 3D ADSC and AC constructs cultured in chondrogenic medium at the end of the culture period were assessed by DMA (Fig 9). Natural calf and chicken articular cartilage samples were also measured under the same assay conditions, allowing us to compare these tissues with the synthetic constructs after 4 weeks of culture. To provide a complete profile of the viscoelastic behavior of the samples, different parameters were studied: storage modulus (G’), loss modulus (G”), complex modulus (G*) and tan(delta). The elastic component (represented by G’) showed a different profile between cell types. In the case of ADSC constructs, the values of G’ were comparable to chicken and calf articular cartilage (Fig 9A). In contrast, AC constructs displayed significantly lower G’ values than did the native cartilage samples (Fig 9E). Moreover, no significant differences were detected between scaffold types. The viscous component (G”) and the complex modulus (G*) for both cell types showed a more similar tendency than G’ between 3D constructs and cartilage controls (Fig 9B, 9C, 9F and 9G). However, all samples presented with G’ values that were much higher than the G” values, indicating that the constructs were more elastic than viscous. Tan(delta) values, which gives an idea of the full mechanical response of the material, showed that all 3D constructs were comparable to chicken cartilage and differ from calf cartilage (Fig 9D and 9H). Thus, we conclude that the mechanical behavior of our ADSC constructs is more similar to chicken and calf native articular cartilage (Fig 9A–9D) than is the mechanical behavior of our AC constructs (Fig 9E–9H).

## Discussion

In this study, CS and Decorin molecules were combined with the self-assembling RAD16-I peptide to develop new scaffolds for CTE applications. RAD16-I hydrogel alone was previously used to support chondrogenesis with different cell types [34,35]. Moreover, RAD16-I was combined with heparin moieties to generate a bi-component scaffold with bioactive signals to promote capillary morphogenesis of endothelial cells and enhance the chondrogenesis of ADSCs and chondrocytes [39,40]. In this work, CS and Decorin molecules were selected based on their ability to mimic the natural ECM of articular cartilage and generate chondro-favorable biochemical cues in the 3D microenvironment. In fact, prior CTE strategies have evaluated the combination of CS with different hydrogel scaffolds, such as chitosan [49], poly(ethylene glycol) (PEG) [50] or collagen type I [51]. Although several studies have explored the influence of CS on chondrogenesis, less is known about the ability of Decorin to guide chondrogenic commitment. Therefore, in the present work, we studied the influence of both CS and Decorin on chondrogenesis in a nanometric 3D system. As in the case of RAD16-I/Heparin composites [39,40], CS and Decorin-based self-assembling scaffolds were generated with a simple mixture of the two components (see Material and Methods). The bi-component scaffolds exhibited structural stability at physiological pH, wherein β–sheet structural characteristics of the self-assembling peptide were maintained (Fig 1). Moreover, CS and Decorin molecules were homogenously distributed in the nanofiber network, as evidenced by toluidine blue staining. We suggest that the hydrophilic, negatively charged nature of CS molecules (and Decorin PGs containing a CS chain) allows the interaction of CS and Decorin molecules with the positive residues of the amphiphilic RAD16-I peptide via the electrostatic interactions that occur during the self-assembling process.

Our TGFβ1 release studies revealed that the RAD/CS composite released more TGFβ1 within the first 24 hours compared to the control scaffold. Although we could not calculate the quantity of TGFβ1 initially bound to the hydrogel, we believe that this difference was likely due to a differential GF binding affinity to the scaffold. Thus, we reason that more TGFβ1 could be initially bounded to the RAD/CS scaffold compared to the RAD16-I scaffold. This slow release process suggests that TGFβ1 may be bound to the carbohydrate moiety and presented to the cell’s surface GF receptor, thereby promoting a signal cascade comparable to that which occurs physiologically. The development of this type of bi-component scaffold (structural-signaling integrated) could be applied towards deconstructing the complex signaling network to which cells are exposed during differentiation (ADSCs) or reengagement of lineage commitment (de-differentiated ACs). In fact, the present work was aimed at promoting cartilage tissue development *in vitro* using the above-mentioned paradigm: two cell types (multipotent ADSCs and dedifferentiated ACs) in a 3D bi-component scaffold with chondrogenic induction media (i.e., containing TGFβ1).

Cells embedded in the nanometric RAD16-I scaffold experience a truly 3D environment, as demonstrated in previous studies [29,30,35,52], where they can elongate, interconnect with neighboring cells and matrix, proliferate and extend different cellular processes. Hence, this 3D culture system models the *in vivo* environment, and depending on the conditions provided (culture medium, cell type, scaffold functionalization, etc.), could evolve into different cellular microenvironments [53]. In particular, our work revealed differences between the behavior of ADSCs and ACs cultured in the same scaffolds. ADSCs became elongated and formed a cellular network only under chondrogenic conditions, whereas ACs appeared elongated in all culture conditions (Fig 2). However, ACs were more compact and established connections in both expansion and chondrogenic medium. Indeed, it appears that the control medium did not promote cellular spreading and interconnectivity. For both cell types, the diameter of constructs cultured in control medium was reduced by only a small amount from the initial state. In contrast, constructs cultured in chondrogenic medium underwent a significant scaffold condensation during the culture timeframe, resulting in a compacted structure after 4 weeks. This morphological change was likely prompted by forces exerted by the cells and the matrix, a remodeling process stimulated by the chondro-inductive factors contained in the medium (e.g., TGFβ1).

Differences in cell viability were also observed between cell types. ADSCs were only alive after 4 weeks of culture under chondrogenic conditions or in the presence of the heparin scaffold (Figs 3 and 4). In contrast, ACs were found to be viable in all experimental conditions, but their relative viability in control medium was reduced compared to expansion and chondrogenic media. Therefore, we suggest that the presence of GAGs in the scaffold enhanced cell viability and their general performance during the 4 weeks of culture (Figs 3B and 4B).

The expression of important chondrogenic markers, including *COL2*, *SOX9* and *ACAN*, in 3D ADSC constructs was increased compared to monolayer cultures (Fig 6). Similarly, in AC constructs, the expression of these markers was stimulated under chondrogenic conditions and was decreased in expansion medium. Therefore, the combination of scaffold GAGs and chemical inducers present in chondrogenic medium led to the activation of signaling pathways that are important for the chondrogenic commitment. The mechanism underlying this activation, however, remains poorly understood. At the protein level, western blot results revealed a possible increased COL1 maturation process in 3D cultures of both cell types when compared to the COL1 detected in 2D cultures [54]. In particular, a pattern of four main bands was detected (220 kDa, 180 kDa, 160 kDa and 130 kDa). The final mature COL1 product corresponds to the lower molecular weight band (Fig 7). Moreover, mature COL1 was predominant in constructs cultured in chondrogenic medium, suggesting that COL1 was only properly processed in 3D constructs cultured in chondrogenic medium and, therefore, contributes to the formation of a more physiologically representative matrix. Importantly, the expression of COL2 was confirmed in 3D cultures under chondrogenic conditions and was not previously detected in 2D cultures. Additionally, toluidine blue staining revealed the production of GAGs by both 3D ADSCs and 3D ACs cultured in chondrogenic medium (Fig 8). These results indicate the synergistic effect of the 3D culture system and the chondrogenic medium in stimulating the production of collagen and GAG components of the ECM, which could play an important role in matrix remodeling. Moreover, these constructs did not mineralize the scaffold, as indicated by von Kossa staining, suggesting that 3D constructs did not undergo cartilage hypertrophy during the culture period.

Finally, mechanical characterization showed that the viscoelastic behavior of ADSC constructs more closely resembled native cartilage than did the viscoelastic behavior of AC constructs (Fig 9). In both cell types, no significant differences were detected between CS- or Decorin-scaffold constructs. This finding suggests that the initial composition of the hydrogels did not influence the resultant mechanical properties of the constructs. As previously mentioned, constructs cultured in chondrogenic medium experienced a contraction process during the culture period that resulted in a compacted structure (Fig 2) with mechanical properties that changed from day 0 to the end of the culture period. In contrast, the diameter of constructs cultured in control medium was reduced by only a small amount from the initial state, and the mechanical properties at the end of the culture differed greatly from those of the chondrogenic constructs. Control constructs formed softer structures that could not be measured under the same conditions as chondrogenic constructs, owing to the disparity in mechanical properties among construct types. Similarly, the initial mechanical characteristics of the RAD16-I scaffold alone could not be measured under the same conditions as the chondrogenic constructs due to the soft nature of the peptide. However, previous studies report that the initial peptide concentration at which cells were embedded (0.15% w/v RAD16-I) corresponds to 100 Pa [36]. This soft microenvironment and the nature of the hydrogel allows cells to freely migrate, interconnect and extend different cellular processes in a dynamic and permissiveness milieu [27]. Therefore, as a consequence of the matrix remodeling process by the cells, constructs evolve into stiffer structures which better mimic the mechanical properties of native cartilage.

In summary, the present study reports promising results for different chondrogenic scenarios, revealing the functionality and versatility of novel bi-component scaffolds, depending on the conditions provided. Moreover, the availability and the ease of preparation of our novel biomaterials make them suitable for future *in vivo* applications.
